# Perceived stress and health behaviors among nurses in Poland and Slovakia: a cross-sectional comparative study

**DOI:** 10.3389/fpubh.2026.1976809

**Published:** 2026-10-26

**Authors:** Katarzyna Tomaszewska, Bożena Majchrowicz, Ľudmila Matulníková, Helena Kadučáková, Katarína Zrubáková, Alicja Kłos, Krystyna Kowalczuk

**Affiliations:** 1 Department of Nursing, Institute of Health Protection, The Bronisław Markiewicz Academy of Applied Sciences, Jarosław, Poland; 2 Collegium Medicum, Institute of Nursing, University of Rzeszów, Rzeszów, Poland; 3 St. Elizabeth University of Health and Social Work, Bratislava, Slovakia; 4 Department of Nursing, Faculty of Health Sciences, Catholic University in Ružomberok, Ružomberok, Slovakia; 5 Department of Health Care Sciences, Faculty of Humanities, Tomas Bata University in Zlin, Zlin, Czechia; 6 Department of Integrated Medical Care, Medical University of Białystok, Białystok, Poland

**Keywords:** health behavior inventory, health behaviors, nurses, occupational stress, PSS-10

## Abstract

**Introduction:**

Occupational stress represents a substantial burden on nurses’ physical and mental health and has been associated with less favorable health behaviors. Despite the large body of research on occupational stress, there is still a lack of comparative studies examining the relationship between perceived stress levels and health behaviors among nurses in Central European countries. The aim of this study was to assess the relationship between perceived stress levels and health behaviors among nurses employed in healthcare institutions in Poland and Slovakia, as well as to examine the associations of selected sociodemographic and occupational factors with the variables analyzed.

**Materials and methods:**

A multicenter cross-sectional study was conducted involving 307 nurses, including 145 from Poland and 162 from Slovakia. An original survey questionnaire, the Perceived Stress Scale (PSS-10), and the Health Behavior Inventory (HBI) were utilized. Statistical analysis was performed using the Mann–Whitney U test and Spearman’s rank correlation coefficient. The level of statistical significance was set at *p* < 0.05.

**Results:**

No significant differences were found between nurses from Poland and Slovakia in perceived stress levels (*p* = 0.551). Nurses from Slovakia had significantly higher scores for overall health behaviors, positive mental attitude, and preventive behaviors (*p* < 0.05), while the difference in health practices was at the threshold of statistical significance (*p* = 0.049; Monte Carlo *p* = 0.050). In both groups, significant negative correlations were found between perceived stress levels and health behaviors (ρ ranging from −0.179 to −0.427; *p* < 0.05). The study groups differed in terms of age and length-of-service distributions, which should be taken into account when interpreting between-group comparisons.

**Conclusion:**

Higher levels of perceived stress were associated with less favorable health behaviors in both groups of nurses. Despite similar levels of perceived stress, nurses from Slovakia achieved higher overall health behavior scores than nurses from Poland. Given the cross-sectional design of the study and the sociodemographic and occupational differences between the groups, between-country comparisons should be interpreted with caution. The findings highlight the importance of interventions aimed at reducing occupational stress and promoting health-enhancing behaviors among nursing staff.

## 1 Introduction

Occupational stress is recognized as one of the major challenges facing contemporary healthcare systems and remains associated with both the well-being of healthcare professionals and the quality and safety of care provided. Among healthcare professionals, nurses constitute a particularly vulnerable group, whose daily work involves high responsibility for the lives and health of patients, the necessity to make rapid clinical decisions, intense emotional burden, and frequent exposure to suffering, severe illness, and death. Additional sources of stress include staff shortages, an aging patient population, the growing complexity of care, shift work, and increasing organizational and administrative demands. Prolonged exposure to occupational stress is associated with an elevated risk of deterioration in mental and physical health, the development of occupational burnout, decreased job satisfaction, an increase in medical errors, sickness absence, and attrition from the profession, which poses a significant threat to both the personnel and the functioning of the healthcare system (1–7).

Perceived stress refers to the subjective evaluation of the extent to which an individual views environmental demands as exceeding their coping capacities. According to Lazarus and Folkman’s transactional model of stress, the stress response is not solely a consequence of a stressor but results from a process of cognitive appraisal of the situation and the available resources to manage it (1). Consequently, individuals operating under similar occupational conditions may experience different levels of stress, depending on their individual psychological resources, professional experience, social support, and utilized coping strategies. One of the most frequently used tools to assess this phenomenon is the Perceived Stress Scale (PSS) developed by Cohen et al., which enables the evaluation of the degree to which life events are perceived as unpredictable, uncontrollable, and overwhelming (2).

Health behaviors are among the fundamental determinants of health and include conscious actions undertaken by individuals to maintain, strengthen, or improve their health. In Pender’s Health Promotion Model, health-promoting behaviors are considered within the broader context of individual characteristics and experiences, as well as behavioral-specific cognitions and affect, including perceived benefits and barriers, self-efficacy, and interpersonal and situational influences (8). In the present study, this model served only as a theoretical context for understanding health-promoting behaviors rather than as a framework subjected to empirical testing. Self-reported health behaviors were assessed using the Health Behavior Inventory (HBI), whereas other constructs of Pender’s model, such as perceived benefits and barriers or self-efficacy, were not measured. Among nurses, health behaviors are particularly important because they influence not only their psychophysical condition and ability to perform professional duties, but also the credibility of the health-related recommendations they provide to patients (9–12).

Research findings among nurses indicate that despite a high level of knowledge regarding health promotion, many do not achieve the recommended level of physical activity, lead a sedentary lifestyle, eat irregularly, experience sleep deprivation, and face difficulties in maintaining a work-life balance. Furthermore, a study by Gieniusz-Wojczyk et al. (13) demonstrated that the health behaviors of nurses are related to personality traits, particularly Type D personality, highlighting the significance of psychological factors in shaping the lifestyle of this professional group. In turn, Bartosiewicz and Łuszczki (14), as well as Bartosiewicz et al. (15), reported a high prevalence of sedentary behaviors among Polish nurses, while more recent studies by the same group of authors highlighted the frequent occurrence of unhealthy dietary habits and abnormalities in body composition. These findings suggest that despite their professional preparation to conduct health education, nurses themselves often fail to follow healthy lifestyle recommendations, which may increase the risk of developing chronic diseases and impair their ability to cope with chronic occupational stress. Additionally, it has been shown that a higher level of health behaviors is associated with a better quality of life, greater psychological well-being, and higher job satisfaction. Orszulak et al. demonstrated that nurses exhibiting more favorable health behaviors also achieved higher scores in the assessment of quality of life, confirming the close relationship between lifestyle and the professional functioning of this group (16). Moreover, Ważna et al. found that age, level of education, place of employment, work experience, and selected factors of the work environment constitute significant predictors of nurses’ health behaviors. The results of these studies indicate that assessing health behaviors requires considering both the individual characteristics of the employee and the organizational and environmental determinants that may enhance or limit the adoption of health-promoting actions (17).

Previous studies indicate a complex and potentially bidirectional relationship between perceived stress and health behaviors (18). On the one hand, higher levels of stress have been associated with less favorable health behaviors, such as reduced physical activity, irregular eating patterns, shorter sleep duration, and less frequent engagement in preventive health practices. On the other hand, more favorable health behaviors have been associated with better psychological functioning and greater adaptive capacity. Given the cross-sectional design of the present study, the observed relationships were interpreted solely as associations between variables, without inferring the direction of causality. Contemporary health promotion models suggest that health behaviors are not merely the result of individual choices, but rather emerge from complex interactions between individual characteristics, the work environment, and social and organizational factors. This relationship is particularly important in the nursing profession. Prolonged psychological strain related to professional responsibility, shift work, staff shortages, and high organizational demands may coexist with less favorable lifestyle patterns. Nurses experiencing higher levels of stress have been shown to report insufficient physical activity, poorer sleep quality, unhealthy dietary habits, and less frequent engagement in preventive health behaviors. At the same time, more favorable health behaviors have been associated with greater resilience to stress, better quality of life, and higher job satisfaction (19, 20).

It should be emphasized, however, that the relationship between stress and health behaviors is not uniform across all groups of nurses. Previous studies indicate that its strength and direction may be modified by age, education, work experience, place of employment, type of ward, shift work system, number of employment locations, and the level of organizational support. Increasing attention is also being paid to the significance of psychosocial factors, such as psychological resilience, self-efficacy, Type D personality, and the availability of social support. The research results of Ważna et al. (17) and Gieniusz-Wojczyk et al. (13) confirm that both sociodemographic and psychological factors can significantly influence the adoption of health behaviors by nurses, justifying the need to include them in analyses of the relationship between stress and lifestyle.

Despite the systematic increase in the number of studies concerning the occupational stress of nurses, most of them focus on its relationship with occupational burnout, quality of life, job satisfaction, mental health, or the intention to leave the profession. The relationships between the level of perceived stress and health behaviors, especially with the simultaneous consideration of sociodemographic and occupational factors, are analyzed much less frequently. Previous research conducted in Poland has provided valuable information regarding selected determinants of nurses’ health behaviors; however, it mainly covered single aspects of lifestyle or selected psychological predictors (13–17, 19, 20).

Perceived stress is also associated with the ways in which occupational demands are managed. In the study by Lafraxo et al. (21), problem-focused coping strategies were the most commonly used among nursing staff, while higher levels of stress were associated with more frequent seeking of social support. Previous comparative studies conducted among nurses from Poland and Slovakia, as well as those addressing the broader Central European context, have focused, among other issues, on psychological burden, occupational stress, burnout, and workload (22, 23). To further clarify the research gap, a targeted literature search was conducted in PubMed/MEDLINE, Scopus, and Web of Science using combinations of terms related to nurses, perceived stress, health behaviors, Poland, and Slovakia. No studies were identified that directly compared nurses from Poland and Slovakia with regard to the relationship between perceived stress levels and health behaviors assessed using the PSS-10 and HBI. Therefore, the existing literature does not establish whether the relationship between perceived stress and health behaviors is similar in the two populations. Comparative studies conducted in Central European countries may provide important insights into the associations of sociodemographic and occupational factors with nurses’ functioning. Identifying factors associated with more favorable health behaviors and lower levels of perceived stress may provide a basis for designing interventions aimed at health promotion, prevention of occupational stress, and support of nurses’ well-being. The findings of such studies may be relevant not only to clinical practice, but also to human resource management in healthcare and health policy planning (22–25).

*The aim of this study* was to assess the relationship between perceived stress levels and health behaviors among nurses employed in healthcare institutions in Poland and Slovakia, as well as to examine the associations of selected sociodemographic and occupational factors with the variables analyzed.

Given the exploratory and comparative nature of the study, the following research questions were formulated:

1. Do perceived stress levels and health behaviors differ between nurses from Poland and Slovakia?
2. What is the relationship between perceived stress levels and overall health behaviors, as well as their individual dimensions, in the two study groups?
3. Are selected sociodemographic and occupational factors associated with perceived stress levels and health behaviors among nurses?

## 2 Materials and methods

### 2.1 Research design

The study was designed as a two-center, cross-sectional, comparative observational study conducted using a diagnostic survey method. The aim was to assess the relationship between perceived stress levels and health behaviors among nurses employed in healthcare institutions in Poland and Slovakia, as well as to examine the associations of selected sociodemographic and occupational factors with the variables analyzed.

The study was conducted among nurses employed in two hospitals, one in Poland and one in Slovakia. The Slovak part of the study was carried out in May 2026 among nursing staff employed at a hospital in Bratislava, whereas the Polish part was conducted in April 2026 among nurses employed at a hospital in the Podlaskie Voivodeship. A non-probability convenience sampling method was used. A total of 307 nurses were included in the study, including 145 from Poland and 162 from Slovakia.

The inclusion criteria were: being a practicing nurse, being actively employed in a healthcare institution, being at least 18 years of age, and providing informed consent to participate in the study. The exclusion criteria included incomplete questionnaires and lack of consent to participate.

### 2.2 Research tools

#### 2.2.1 Original survey questionnaire

The questionnaire was developed specifically for the purposes of the present study. The author-designed survey questionnaire was used exclusively to collect participants’ sociodemographic and occupational data, including gender, age, education, length of professional experience, place of employment, marital status, work schedule, having children, and number of overtime hours. The questionnaire was not a psychometric instrument and was not used to measure any construct or to calculate a total score. Therefore, its internal consistency was not assessed. The same set of sociodemographic and occupational questions was used in both countries and was presented to participants in the appropriate language version.

#### 2.2.2 Perceived stress scale (PSS-10)

Perceived stress levels were assessed using the 10-item Perceived Stress Scale (PSS-10) developed by Cohen and Williamson (26). The tool is used to evaluate the degree to which life situations over the past month are perceived as stressful, unpredictable, and difficult to control. Each of the 10 items is rated on a five-point Likert scale (0–4), and the total score ranges from 0 to 40 points. Higher scores indicate a greater level of perceived stress. In the Polish cohort, the Polish adaptation of the scale developed by Juczyński (27) was used, which is characterized by good reliability (Cronbach’s *α* ≈ 0.86). In the Slovak part of the study, a validated Slovak version of the PSS-10 was used, and its psychometric properties had been confirmed in validation studies (28).

#### 2.2.3 Health behavior inventory (HBI)

Health behaviors were evaluated using the Health Behavior Inventory (HBI) authored by Zygfryd Juczyński (29). The tool consists of 24 statements rated on a five-point Likert scale (1–5). It allows for the determination of a general health behavior index (24–120 points) and the assessment of four subscales:

- Proper dietary habits,
- Preventive behaviors,
- Positive mental attitude,
- Health practices.

Higher scores indicate a greater intensity of health-promoting behaviors. In the Polish cohort, the original Polish version of the tool developed by Juczyński was used, which demonstrated good internal consistency (Cronbach’s *α* of approximately 0.85 for the overall score). In the Slovak cohort, a Slovak linguistic version of the Health Behavior Inventory was applied, prepared in accordance with the forward-backward translation procedure by two independent translators. The final version of the questionnaire was agreed upon by the Polish-Slovak research team to ensure linguistic and semantic equivalence with the original version of the tool. Due to the lack of published psychometric validation of the Slovak version of the HBI, the tool was used as a linguistic adaptation, and the obtained results were interpreted with this limitation in mind.

### 2.3 Participants

The study was conducted among nurses employed in healthcare facilities in Poland and Slovakia. The Slovak part of the study was carried out among nursing staff employed in a hospital in Bratislava in May 2026. The research in Poland was conducted in April 2026 among nurses employed in hospitals within the Podlaskie Voivodeship. In total, 307 nurses were included in the study, comprising 145 from Poland and 162 from Slovakia. The inclusion criteria for the study were: practicing the nursing profession, active employment in a healthcare facility, being at least 18 years of age, and providing informed consent to participate in the study. Exclusion criteria included incomplete questionnaires and a lack of consent to participate.

### 2.4 Data collection procedure

The study was conducted using paper-based questionnaires. At each of the two study sites, 180 questionnaires were provided to the study coordinators for distribution among nurses who met the inclusion criteria. Participation in the study was voluntary, anonymous, and uncompensated. Before participation, respondents were informed about the purpose of the study, the study procedures, and their right to withdraw at any stage without providing a reason. After providing informed consent, participants completed the questionnaires independently. Only fully completed questionnaires were included in the analysis: 145 from Poland and 162 from Slovakia, representing 80.6 and 90.0%, respectively, of the questionnaires distributed at each study site. The remaining questionnaires (*n* = 53) were returned blank or incomplete and were excluded from the analysis. Overall, 307 of the 360 distributed questionnaires were included in the analysis, corresponding to an overall response rate of 85.3%. As only fully completed questionnaires were included in the analyses, no missing-data imputation methods were applied.

### 2.5 Ethical procedure

The study is part of a broader research project concerning the psychosocial workload of nurses. It was conducted in accordance with the principles of the Declaration of Helsinki. Approval was obtained from the Bioethics Committee of the Medical University of Białystok (APK.002.493.2025) and the Bioethics Committee of St. Elizabeth University of Health and Social Work in Bratislava (No. 18/2026). All participants provided informed consent to participate in the study.

### 2.6 Statistical analysis

Statistical analyses were performed using IBM SPSS Statistics. The characteristics of the study population were summarized using frequencies and percentages for categorical variables and means, standard deviations, medians, minimum values, and maximum values for continuous variables. The Mann–Whitney U test was used to compare outcomes between nurses from Poland and Slovakia because the analyzed variables did not meet the assumptions of normality.

Associations between perceived stress levels, health behaviors, and selected sociodemographic and occupational variables were assessed using Spearman’s rank correlation coefficient (rho), which was also used to determine the direction and strength of the observed associations. For analyses in which a Monte Carlo *p*-value was calculated, this value was used as the basis for assessing statistical significance. The primary analyses included comparisons of perceived stress levels and overall health behavior scores between the two study groups, as well as assessment of the association between the PSS-10 score and the overall HBI score. Analyses of the individual HBI dimensions and their associations with sociodemographic and occupational characteristics were considered exploratory. Given the exploratory nature of these analyses, no formal adjustment for multiple comparisons was applied. The results of the exploratory analyses were therefore interpreted with caution, taking into account the increased risk of Type I error. Statistical significance was set at *p* < 0.05.

## 3 Results

### 3.1 Characteristics of the study group

The study included 307 nurses, comprising 145 from Poland and 162 from Slovakia (Table 1). The demographic structure of the two groups differed primarily in terms of age and professional experience. Among the nurses from Poland, individuals under 25 years of age (65.5%) and those working in the profession for less than a year (63.4%) predominated. Conversely, in the Slovak group, the most numerous were individuals aged 35–44 years (32.7%) and nurses with 1 to 5 years of work experience (43.2%).

**Table 1:** Sociodemographic and occupational characteristics of the surveyed nurses from Poland and Slovakia.

Variable	Category	Poland (*n* = 145) *n* (%)	Slovakia (*n* = 162) *n* (%)	Total (*n* = 307) *n* (%)
Age (years)	<25	95 (65.5)	33 (20.4)	128 (41.7)
	25–34	7 (4.8)	31 (19.1)	38 (12.4)
	35–44	22 (15.2)	53 (32.7)	75 (24.4)
	45–54	16 (11.0)	39 (24.1)	55 (17.9)
	≥55	5 (3.4)	6 (3.7)	11 (3.6)
Gender	Female	135 (93.1)	150 (92.6)	285 (92.8)
	Male	10 (6.9)	12 (7.4)	22 (7.2)
Nursing education	Secondary, Bachelor’s, Master’s	139 (95.9)	146 (90.1)	285 (92.8)
	Postgraduate studies, specialization	6 (4.1)	6 (3.7)	12 (3.9)
	Other	0 (0.0)	10 (6.2)	10 (3.3)
Work experience (years)	<1	92 (63.4)	31 (19.1)	123 (40.1)
	1–5	7 (4.8)	70 (43.2)	77 (25.1)
	6–10	8 (5.5)	28 (17.3)	36 (11.7)
	11–20	23 (15.9)	17 (10.5)	40 (13.0)
	21–30	7 (4.8)	12 (7.4)	19 (6.2)
	31–40	7 (4.8)	4 (2.5)	11 (3.6)
	>40	1 (0.7)	0 (0.0)	1 (0.3)
Place of employment	Internal medicine ward	81 (55.9)	26 (16.0)	107 (34.9)
	Surgical ward	43 (29.7)	23 (14.2)	66 (21.5)
	Hospital emergency department	21 (14.5)	4 (2.5)	25 (8.1)
	Pediatrics	0 (0.0)	9 (5.6)	9 (2.9)
	Other ward	0 (0.0)	100 (61.7)	100 (32.6)
Marital status	Single	90 (62.1)	66 (40.7)	156 (50.8)
	Married	29 (20.0)	79 (48.8)	108 (35.2)
	Divorced	25 (17.2)	14 (8.6)	39 (12.7)
	Widowed	1 (0.7)	3 (1.9)	4 (1.3)
Work system	Single-shift 8-h	26 (17.9)	43 (26.5)	69 (22.5)
	Single-shift 12 h	39 (26.9)	19 (11.7)	58 (18.9)
	Shift work (day/night)	44 (30.3)	85 (52.5)	129 (42.0)
	Shift work (rotations)	32 (22.1)	9 (5.6)	41 (13.4)
	Other	4 (2.8)	6 (3.7)	10 (3.3)
Having children	One child	32 (22.1)	28 (17.3)	60 (19.5)
	Two children	8 (5.5)	52 (32.1)	60 (19.5)
	Three or more children	15 (10.3)	15 (9.3)	30 (9.8)
	No children	90 (62.1)	67 (41.4)	157 (51.1)
Number of overtime hours per month	0	41 (28.3)	28 (17.3)	69 (22.5)
	1–10	52 (35.9)	56 (34.6)	108 (35.2)
	11–20	23 (15.9)	47 (29.0)	70 (22.8)
	21–30	16 (11.0)	25 (15.4)	41 (13.4)
	31–40	9 (6.2)	6 (3.7)	15 (4.9)
	>40	4 (2.8)	0 (0.0)	4 (1.3)

The “other ward” category in the Slovak group included neurology, geriatrics, and rheumatology wards.

In both studied populations, the vast majority were women (over 92%), and the predominant level of education was a bachelor’s or master’s degree in nursing. Differences were also observed regarding the place of employment. Nurses from Poland most frequently worked in internal medicine wards, surgical wards, and hospital emergency departments, whereas the Slovak group was dominated by individuals employed in other hospital wards and pediatric wards Table 2.

**Table 2:** Comparison of the level of perceived stress and health behaviors among nurses from Poland and Slovakia.

Group		Positive mental attitude (1–5)	Preventive behaviors (1–5)	Proper dietary habits (1–5)	Health practices (1–5)	Total HBI (24–120)	PSS-10 perceived stress (0–40)
Slovakia	Mean	3.41	3.53	3.14	2.95	78.22	20.70
	Median	3.50	3.67	3.17	3.00	79.00	21.00
	Mean rank	177.57	178.43	159.25	163.42	171.97	156.85
	*N*	162	162	162	162	162	162
	Standard deviation	0.84	0.70	0.81	0.79	15.74	4.88
	Minimum	1.00	1.50	1.17	1.17	32.00	3.00
	Maximum	5.00	5.00	5.00	4.67	117.00	33.00
Poland	Mean	2.97	3.08	3.08	2.78	71.48	20.37
	Median	3.17	3.00	3.00	2.83	71.00	21.00
	Mean rank	127.67	126.71	148.13	143.48	133.92	150.81
	*N*	145	145	145	145	145	145
	Standard deviation	0.78	0.83	0.76	0.77	15.48	5.48
	Minimum	1.17	1.50	1.50	1.33	42.00	8.00
	Maximum	4.83	5.00	5.00	4.67	108.00	35.00
Total	Mean	3.20	3.32	3.11	2.87	75.04	20.54
	Median	3.33	3.33	3.17	2.83	76.00	21.00
	Mean rank	307	307	307	307	307	307
	*N*	0.84	0.80	0.79	0.79	15.95	5.17
	Standard deviation	1.00	1.50	1.17	1.17	32.00	3.00
	Minimum	5.00	5.00	5.00	4.67	117.00	35.00
Manna-Whitney U		Maximum	7787.50	10894.50	10219.50	8834.00	11283.00
*p*		< 0.001	< 0.001	0.273	0.049	< 0.001	0.551
*p* (Monte Carlo)		< 0.001	< 0.001	0.275	0.050	0.001	0.553

Among the respondents from Poland, single and divorced individuals were more common, whereas in the Slovak group, a larger percentage consisted of married individuals. Nurses from Slovakia more often worked in a shift system comprising day and night shifts, as well as in an eight-hour single-shift system, whereas in Poland, a twelve-hour single-shift system and a rotating system were more frequently reported.

Having children was more frequently reported by nurses from Slovakia, while childlessness more often applied to respondents from Poland. Furthermore, nurses from Poland more frequently indicated a lack of monthly overtime hours; however, they simultaneously reported working more than 30 h of overtime per month more often than respondents from Slovakia.

### 3.2 Comparison of health behaviors and perceived stress levels in the study groups

The mean score of the general health behavior index (HBI) was higher in the group of nurses from Slovakia than in the Polish group (78.22 ± 15.74 vs. 71.48 ± 15.48 points). A similar trend was observed in the majority of the analyzed HBI subscales. Nurses from Slovakia obtained higher mean scores in terms of positive mental attitude (3.41 ± 0.84 vs. 2.97 ± 0.78), preventive behaviors (3.53 ± 0.70 vs. 3.08 ± 0.83), and health practices (2.95 ± 0.79 vs. 2.78 ± 0.77). Conversely, the mean values regarding proper dietary habits were similar in both groups, amounting to 3.14 ± 0.81 in the Slovak group and 3.08 ± 0.76 in the Polish group, respectively.

The mean level of perceived stress assessed using the PSS-10 scale was comparable in both studied populations and amounted to 20.70 ± 4.88 points among nurses from Slovakia and 20.37 ± 5.48 points among nurses from Poland. Comparative analysis using the Mann–Whitney *U* test showed that nurses from Slovakia had significantly higher scores for positive mental attitude, preventive behaviors, and the overall Health Behavior Inventory (HBI) score. For health practices, the result was at the threshold of statistical significance (*p* = 0.049; Monte Carlo *p* = 0.050) and should therefore be interpreted with caution. No significant differences were found between the two groups in terms of proper eating habits or perceived stress levels measured using the PSS-10.

### 3.3 Relationship between the level of perceived stress and health behaviors

Spearman’s rank correlation analysis revealed significant negative relationships between the level of perceived stress and health behaviors in both the Polish and Slovak groups of nurses (Table 3). This indicates that as the level of perceived stress increased, health behaviors deteriorated across all analyzed dimensions of the HBI.

**Table 3:** Correlations between the level of perceived stress and health behaviors among nurses from Poland and Slovakia.

	Group	Health behaviors	Statistical parameter	PSS-10 perceived stress (0–40)
Spearman’s rho	Slovakia *N* = 162	Positive mental attitude (1–5)	Correlation coefficient	−0.320**
			Significance (two-tailed)	< 0.001
		Preventive behaviors (1–5)	Correlation coefficient	−0.179*
			Significance (two-tailed)	0.023
		Proper dietary habits (1–5)	Correlation coefficient	−0.261**
			Significance (two-tailed)	0.001
		Health practices (1–5)	Correlation coefficient	−0.293**
			Significance (two-tailed)	< 0.001
		Total HBI (24–120)	Correlation coefficient	−0.351**
			Significance (two-tailed)	< 0.001
	Poland *N* = 145	Positive mental attitude (1–5)	Correlation coefficient	−0.427**
			Significance (two-tailed)	< 0.001
		Preventive behaviors (1–5)	Correlation coefficient	−0.327**
			Significance (two-tailed)	< 0.001
		Proper dietary habits (1–5)	Correlation coefficient	−0.238**
			Significance (two-tailed)	0.004
		Health practices (1–5)	Correlation coefficient	−0.391**
			Significance (two-tailed)	< 0.001
		Total HBI (24–120)	Correlation coefficient	−0.427**
			Significance (two-tailed)	< 0.001

**Correlation is significant at the 0.01 level (two-tailed). *Correlation is significant at the 0.05 level (two-tailed).

In the Slovak group, the correlation coefficients demonstrated weak to moderate strength, ranging from *ρ* = −0.179 to *ρ* = −0.351. The strongest relationship was observed between the stress level and the general health behavior index (*ρ* = −0.351; *p* < 0.001), while the weakest was between the stress level and preventive behaviors (*ρ* = −0.179; *p* = 0.023).

In the Polish group, all analyzed associations were also negative and statistically significant, with correlation coefficients ranging from *ρ* = −0.238 to *ρ* = −0.427. The strongest correlations were observed between perceived stress levels and positive mental attitude, as well as between perceived stress levels and the overall health behavior score (*ρ* = −0.427 in both cases; *p* < 0.001). These findings indicate that higher levels of perceived stress were associated with less favorable health behaviors.

### 3.4 Relationship between selected sociodemographic and occupational factors, perceived stress levels, and health behaviors

Correlation analysis revealed different patterns of associations in the two study groups (Table 4). Among nurses from Slovakia, no significant associations were found between either age or length of professional experience and perceived stress levels or health behaviors (all *p* > 0.05). The only significant association was observed between the number of overtime hours worked and health practices (*ρ* = −0.166; *p* = 0.035), indicating that a greater number of overtime hours was associated with lower health practice scores.

**Table 4:** Correlations of selected sociodemographic and occupational factors with the level of perceived stress and health behaviors among nurses from Poland and Slovakia.

	Group	Analyzed variable		Age	Nursing work experience	Monthly overtime hours
Spearman’s rho	Slovakia *N* = 162	PSS-10 perceived stress (0–40)	Correlation coefficient	−0.142	0.044	0.050
			Significance (two-tailed)	0.071	0.577	0.531
		Positive mental attitude (1–5)	Correlation coefficient	0.127	0.090	−0.038
			Significance (two-tailed)	0.108	0.256	0.633
		Preventive behaviors (1–5)	Correlation coefficient	0.036	−0.031	0.092
			Significance (two-tailed)	0.646	0.698	0.243
		Proper dietary habits (1–5)	Correlation coefficient	0.151	0.037	−0.011
			Significance (two-tailed)	0.055	0.642	0.891
		Health practices (1–5)	Correlation coefficient	0.138	0.064	−0.166*
			Significance (two-tailed)	0.079	0.420	0.035
		Total HBI (24–120)	Correlation coefficient	0.141	0.055	−0.070
			Significance (two-tailed)	0.073	0.489	0.377
	Poland *N* = 145	PSS-10 perceived stress (0–40)	Correlation coefficient	0.123	0.127	0.169*
			Significance (two-tailed)	0.142	0.129	0.043
		Positive mental attitude (1–5)	Correlation coefficient	−0.336**	−0.297**	−0.373**
			Significance (two-tailed)	< 0.001	< 0.001	< 0.001
		Preventive behaviors (1–5)	Correlation coefficient	−0.370**	−0.371**	−0.431**
			Significance (two-tailed)	< 0.001	< 0.001	< 0.001
		Proper dietary habits (1–5)	Correlation coefficient	−0.190*	−0.149	−0.310**
			Significance (two-tailed)	0.022	0.074	0.000
		Health practices (1–5)	Correlation coefficient	−0.399**	−0.383**	−0.398**
			Significance (two-tailed)	< 0.001	< 0.001	< 0.001
		Total HBI (24–120)	Correlation coefficient	−0.396**	−0.367**	−0.455**
			Significance (two-tailed)	< 0.001	< 0.001	< 0.001

**Correlation is significant at the 0.01 level (two-tailed). *Correlation is significant at the 0.05 level (two-tailed).

In the Polish group, a substantially greater number of significant associations was observed. Older age was associated with lower scores for positive mental attitude, preventive behaviors, proper eating habits, health practices, and the overall health behavior score. Similar negative associations were found for length of professional experience, with the exception of proper eating habits, for which no significant correlation was observed. The strongest associations were found between the number of overtime hours worked and preventive behaviors (*ρ* = −0.431; *p* < 0.001), the overall health behavior score (*ρ* = −0.455; *p* < 0.001), and health practices (*ρ* = −0.398; *p* < 0.001). In addition, a greater number of overtime hours was associated with higher perceived stress levels (*ρ* = 0.169; *p* = 0.043).

In summary, nurses from Slovakia had significantly higher scores for overall health behaviors and most of their individual dimensions, whereas perceived stress levels did not differ significantly between the two study groups. In both groups, significant negative associations were observed between perceived stress levels and health behaviors. The correlation coefficients differed between the Polish and Slovak samples; however, differences in the strength of these associations between the groups were not formally tested statistically.

## 4 Discussion

The present study provides new data on the relationship between perceived stress levels and health behaviors among nurses employed in Poland and Slovakia. A significant negative association was observed between perceived stress levels and all analyzed dimensions of health behaviors in both study populations. This indicates that higher levels of perceived stress were associated with less frequent engagement in health-promoting behaviors, including proper eating habits, preventive behaviors, positive mental attitude, and health practices. At the same time, nurses from Slovakia achieved higher overall health behavior scores than nurses from Poland, despite comparable levels of perceived stress. This finding suggests that perceived stress alone does not fully account for the observed differences in health behaviors. However, when interpreting between-group differences, the distinct sociodemographic and occupational characteristics of the two samples, particularly age and length of professional experience, should be taken into account. Because no multivariable analysis was performed, it is not possible to determine to what extent the observed differences were associated with country group membership and to what extent they reflected differences in sample characteristics.

The findings are consistent with the current body of evidence on the relationship between chronic occupational stress and nurses’ functioning. Numerous studies indicate that work-related stress is one of the major factors adversely affecting nurses’ psychological well-being and limiting engagement in health-promoting behaviors. A systematic review and meta-analysis by Woo et al. showed that chronic stress and burnout affect a substantial proportion of nurses worldwide, contributing to poorer occupational functioning and quality of life, as well as increasing the risk of sickness absence, staff turnover, and reduced quality of patient care (30). Similar conclusions were reported by Kelly et al., who emphasized that deterioration in nurses’ psychological well-being has direct implications for patient safety, quality of care, and the functioning of healthcare organizations as a whole (31).

In recent years, increasing attention has been paid to the relationship between occupational stress and health behaviors among healthcare professionals. A meta-analysis by Choi et al. demonstrated that high levels of stress are associated with a greater prevalence of unfavorable health behaviors, including unhealthy dietary habits, reduced physical activity, sleep disturbances, and less frequent engagement in preventive health practices (12). The findings of the present study are consistent with these observations and indicate that this relationship is also present among nurses in Poland and Slovakia. Importantly, the study adds further evidence by showing that, despite comparable levels of perceived stress, health behavior levels may differ between countries. This may point to the potential role of other factors that were not assessed in the present study. An additional contribution of the study is the observation that health behavior levels may differ between study groups even when perceived stress levels are comparable. However, the causes of these differences cannot be determined on the basis of the present study and require further investigation that takes into account individual, occupational, organizational, and environmental characteristics.

A particularly noteworthy finding was the absence of significant differences in perceived stress levels between nurses from Poland and Slovakia despite differences in health behavior levels. Similar PSS-10 scores, however, do not allow conclusions to be drawn regarding similarities in the work environment in the two countries, as work environment characteristics were not directly assessed in the present study. Previous studies conducted among nurses in Central Europe, including those by Kadučáková et al. and Dębska et al. (22, 23), have likewise indicated the presence of psychological burden in this occupational group.

One possible direction for interpreting the observed differences in health behaviors involves organizational characteristics of the work environment. Previous studies suggest that health-promoting behaviors among healthcare professionals may be associated with factors such as organizational culture, management style, social support, and the ability to maintain work–life balance. Kiptulon et al. (10) highlighted the importance of a positive organizational culture in limiting the adverse consequences of occupational stress and supporting health-promoting behaviors, while Wei et al. (32) emphasized the role of teamwork, professional autonomy, and managerial support in nurses’ psychological well-being and quality of care. The importance of the broader occupational context is also supported by recent literature reviews in which workload, occupational stress, working conditions, and organizational factors are among the main domains examined in relation to burnout and well-being among healthcare workers (33). These factors were not directly assessed in the present study; therefore, it cannot be concluded that they explain the differences observed between nurses from Poland and Slovakia. Their potential role should be regarded as a hypothesis requiring verification in future research.

The findings are also consistent with studies emphasizing the importance of personal resources in coping with stress. Rębak et al. reported that nurses who used problem-focused coping strategies and demonstrated better emotional control were more likely to engage in health-promoting behaviors and were less likely to experience occupational burnout (20). Similar observations were reported by Merklinger-Soma et al., who identified psychological resilience, self-efficacy, and optimism as important predictors of health-promoting behaviors among nurses (34). These resources were not assessed in the present study; therefore, their potential role in explaining the observed differences between the study groups cannot be determined on the basis of the present findings. Including these factors in future studies could broaden the interpretation of the relationship between stress and health behaviors.

The present findings are also consistent with Pender’s Health Promotion Model, according to which health behaviors result from the interaction of individual factors, environmental influences, and prior experiences (5). In the present study, perceived stress and health behaviors were assessed, whereas other components of the model, such as perceived benefits and barriers, self-efficacy, and interpersonal and situational influences, were not directly measured. Therefore, Pender’s model provides a theoretical framework for interpreting the observed associations but was not fully tested in the present study.

The analysis of associations between selected sociodemographic and occupational characteristics and health behaviors revealed different patterns in the two study groups. In the Polish group, older age and longer professional experience were associated with lower scores in selected dimensions of health behaviors. In the Slovak group, fewer such associations were observed. However, because correlation coefficients were not formally compared between the groups, it cannot be concluded that these associations were significantly stronger in one study population than in the other. In addition, the cross-sectional design and the absence of multivariable analysis do not allow the mechanisms underlying the observed associations to be determined. These findings should therefore be regarded as exploratory and require confirmation in future studies incorporating a broader range of sociodemographic, occupational, and organizational factors. Similar associations have been described in studies among nursing staff, suggesting that professional experience may promote the development of more effective coping strategies and greater attention to health (35, 36).

At the same time, the findings indicate that these associations were less pronounced among Slovak nurses. This may suggest greater homogeneity of health behaviors regardless of age and professional experience, or a greater role of environmental factors than individual characteristics. However, because the study was cross-sectional, the causes of the observed differences cannot be established. Further research incorporating a broader range of organizational, psychosocial, and cultural factors is therefore needed.

The relationship with overtime work was another important aspect of the analysis. The findings indicate that a greater number of overtime hours was associated with less favorable health behaviors, which is consistent with previous reports suggesting that long working hours reduce opportunities for recovery, make it more difficult to maintain healthy eating habits, decrease physical activity, and increase the risk of chronic fatigue and occupational burnout (37, 38). The importance of workload-related demands is also reflected in the study by Lafraxo et al., in which an imbalance between work effort and received rewards was identified among nurses; within the Effort–Reward Imbalance model, occupational demands include, among other factors, time pressure, high job demands, numerous responsibilities, and overtime work (39). This issue is particularly relevant in nursing, where overtime work may coexist with staffing shortages and increased workload.

The findings also have practical implications, indicating the need to consider both perceived stress and health behaviors in interventions aimed at supporting nurses’ health. Health promotion programs for nurses may include health education, development of stress-management skills, and measures supporting the maintenance of favorable health behaviors. In light of previous research, it may also be appropriate to consider the broader work environment, including workload, opportunities for recovery, and work–life balance. Because organizational factors were not directly assessed in the present study, these proposed actions should be regarded as directions derived from integrating the present findings with previous evidence rather than as interventions whose effectiveness was demonstrated in this study. Such a comprehensive approach may support nurses’ health and, according to previous research, may also have implications for quality of care, sickness absence, and staff turnover (30–32).

The strengths of the present study include the use of a uniform methodology in two countries and the application of standardized and widely used instruments to assess perceived stress and health behaviors. This enabled direct comparison of results obtained among nurses from Poland and Slovakia and assessment of the associations between the analyzed variables in both study groups. The use of an identical study procedure at both sites reduced the risk of differences resulting from variations in study implementation.

## 5 Conclusion

The present study showed that higher levels of perceived stress were associated with less favorable health behaviors among nurses in both Poland and Slovakia. Despite comparable levels of perceived stress, nurses from Slovakia achieved higher overall health behavior scores, as well as higher scores for positive mental attitude and preventive behaviors. For health practices, the difference between the groups was at the threshold of statistical significance. The observed differences between the study groups should be interpreted with caution due to differences in their sociodemographic and occupational characteristics and the lack of adjustment for potential confounding factors. The findings highlight the need to consider both perceived stress and health behaviors in health promotion initiatives targeted at nursing staff.

### 5.1 Implications for practice

Despite the indicated limitations, the obtained results make a significant contribution to the knowledge regarding the health of nursing staff. They confirm that a higher level of perceived stress is associated with less frequent engagement in health-promoting behaviors, and simultaneously indicate that a similar level of stress does not necessarily translate to an identical level of health behaviors across different countries. This suggests that, alongside individual psychological resources, organizational and environmental factors may also play a significant role. The results of the study underscore the need to implement comprehensive programs supporting nurses’ health, encompassing both interventions aimed at stress reduction and the creation of a work environment conducive to adopting health-promoting behaviors. Investing in the well-being of nursing staff can yield benefits not only for the employees themselves but also for patients and the entire healthcare system by improving the quality of care, increasing patient safety, and reducing absenteeism and staff turnover.

### 5.2 Limitations of the study

The present study has several methodological limitations that should be considered when interpreting the findings. First, the cross-sectional design precludes determining the direction of the relationship between perceived stress and health behaviors and does not allow causal inferences to be drawn. Therefore, the possibility that the observed associations are bidirectional cannot be excluded.

Another limitation is the use of non-probability sampling and the inclusion of only two hospitals, one in each country. This limits the generalizability of the findings to the broader nursing populations in Poland and Slovakia and introduces a potential risk of selection bias. Furthermore, data were collected through self-report measures, which may have been subject to recall bias and social desirability bias.

An important limitation is the absence of multivariable analyses that would have allowed simultaneous adjustment for potential confounding factors. The study groups differed in terms of age distribution, length of professional experience, place of employment, and selected work-related characteristics. These differences may have influenced the observed findings regarding health behaviors. Consequently, it is not possible to determine conclusively to what extent the differences identified between Polish and Slovak nurses were attributable to country-specific group membership or to differences in the sociodemographic and occupational characteristics of the participants. The findings should therefore be interpreted as unadjusted associations that do not account for potential confounding factors.

An additional limitation is the lack of formal psychometric validation of the Slovak version of the Health Behavior Inventory (HBI), known in Polish as the Inwentarz Zachowań Zdrowotnych (IZZ), and the absence of an assessment of its internal consistency in the study sample. Although a translation and back-translation procedure was applied and the language version was agreed upon by the Polish–Slovak research team, a comprehensive evaluation of the instrument’s psychometric properties and a formal assessment of measurement equivalence between the two language versions were not conducted. This limits confidence in direct comparisons of health behavior scores between the study groups and warrants caution when interpreting the observed differences.

It should also be acknowledged that the study did not include a detailed assessment of selected organizational and psychosocial factors, such as support from supervisors and colleagues, organizational culture, workload, and job satisfaction, which may be associated with both perceived stress and health behaviors. Furthermore, analyses of individual dimensions of health behaviors and sociodemographic and occupational factors were exploratory, and no adjustment for multiple comparisons was applied. This increases the risk of false-positive findings.

Finally, no formal *a priori* sample size calculation was performed, limiting the ability to assess whether the sample sizes were sufficient to detect all investigated associations, particularly those with small effect sizes.

Taken together, these limitations highlight the need for further research involving larger, more representative samples of nurses, using psychometrically validated and measurement-equivalent versions of the research instruments, as well as multivariable analyses to control for potential confounding factors.

## Data Availability

The raw data supporting the conclusions of this article will be made available by the authors, without undue reservation.
